# 1194. Implementation of Methicillin-Resistant *Staphylococcus Aureus* Nasal Swabs as an Antimicrobial Stewardship Tool in a Community Hospital

**DOI:** 10.1093/ofid/ofad500.1034

**Published:** 2023-11-27

**Authors:** Heather D Gibson, Gretchen S Arnoczy, Jayne Lee, Allison Cid

**Affiliations:** FirstHealth of the Carolinas Moore Regional Hospital, Pinehurst, North Carolina; FirstHealth of the Carolinas, Pinehurst, North Carolina; FirstHealth of the Carolinas Moore Regional Hospital, Pinehurst, North Carolina; FirstHealth of the Carolinas Moore Regional Hospital, Pinehurst, North Carolina

## Abstract

**Background:**

Methicillin-resistant *Staphylococcus Aureus* (MRSA) nasal PCRs are an effective stewardship tool in patients diagnosed with pneumonia and implementation has been shown to reduce use of anti-MRSA antibiotics.

**Methods:**

A pre-post interventional study was conducted at FirstHealth Moore Regional Hospital from January 1^st^ to March 31^st^, 2022 (pre-intervention, PRE) and January 1^st^ to March 31^st^, 2023 (post-intervention, PO). Intervention was education by an antimicrobial stewardship (AMS) pharmacist to providers on the use of MRSA nasal PCRs. Primary outcomes were percent change in patients who had PCR ordered PRE and PO and percent patients who had anti-MRSA therapy (vancomycin, VANC) discontinued based on a negative result. Secondary outcomes include time from negative PCR to VANC discontinuation, length of VANC therapy, and number of VANC levels. Inclusion criteria were patients aged > 18 years old hospitalized for community-acquired pneumonia admitted to a medical floor receiving VANC. Exclusion criteria were patients < 18 years of age, pregnant patients, patients admitted to the ICU, or recent history of MRSA.

**Results:**

For primary outcomes, 125 patients were in the PRE group. 42 were on VANC for > 24 hours and 8 had a MRSA nasal swab (6.4%). Of the 8 swabs, 1 patient (12.5%) had VANC discontinued based on a negative PCR. 2 patients were not started on VANC in response to the negative PCR (25%). 112 patients were in the PO group. 32 were on VANC for > 24 hours and 27 had a MRSA nasal swab performed (24.1%). Of the 27 swabs, 14 (51.9%) had VANC discontinued based on a negative PCR. 6 patients were not started on VANC in response to the negative PCR (22.2%). For secondary outcomes in the PRE group, time from negative PCR to discontinuation of VANC was 9 hours 54 minutes (1 patient), average VANC therapy was 4.8 days, and an average of 1.7 VANC levels were drawn. In the PO group, average time from negative PCR to discontinuation of VANC was 18 hours 18 minutes (14 patients), average VANC therapy was 3.4 days, and an average of 1.1 VANC levels were drawn.

**Conclusion:**

Educating providers on the benefits of MRSA nasal PCRs led to a significant increase in number of PCRs orders and an increase in VANC discontinued with negative results. Secondary outcomes show continued room for improvement and potential for cost avoidance.

**Disclosures:**

**Jayne Lee, BSN, MPH, CIC, FAPIC**, Medline: Advisor/Consultant

